# HTLV-1 Tax Tug-of-War: Cellular Senescence and Death or Cellular Transformation

**DOI:** 10.3390/pathogens13010087

**Published:** 2024-01-19

**Authors:** Marcia Bellon, Christophe Nicot

**Affiliations:** Department of Pathology and Laboratory Medicine, University of Kansas Medical Center, 3901 Rainbow Blvd, Kansas City, KS 66160, USA; mbellon@kumc.edu

**Keywords:** HTLV-1, transformation, Tax, NF-kB, senescence, cell cycle, tumor suppressor, DNA damage, genomic instability, apoptosis, telomerase

## Abstract

Human T cell leukemia virus type 1 (HTLV-1) is a retrovirus associated with a lymphoproliferative disease known as adult T cell leukemia/lymphoma (ATLL). HTLV-1 infection efficiently transforms human T cells in vivo and in vitro. The virus does not transduce a proto-oncogene, nor does it integrate into tumor-promoting genomic sites. Instead, HTLV-1 uses a random mutagenesis model, resulting in cellular transformation. Expression of the viral protein Tax is critical for the immortalization of infected cells by targeting specific cellular signaling pathways. However, Tax is highly immunogenic and represents the main target for the elimination of virally infected cells by host cytotoxic T cells (CTLs). In addition, Tax expression in naïve cells induces pro-apoptotic signals and has been associated with the induction of non-replicative cellular senescence. This review will explore these conundrums and discuss the mechanisms used by the Tax viral oncoprotein to influence life-and-death cellular decisions and affect HTLV-1 pathogenesis.

## 1. Introduction

HTLV-1 is the only human retrovirus etiologically linked to cancer [1,2,3]. In vivo and in vitro, the virus preferably infects and induces the proliferation of mature CD3+ CD4+ T cells [4], eventually culminating in the development of T cell leukemia or lymphoma. Clinically, ATLL can present in different forms that differ in disease progression, treatment, and survival outcomes and are referred to as smoldering, chronic, or acute type [5,6,7]. HTLV-I-associated diseases have no cure and the mean survival of acute and lymphoma ATLL patients is 6–10 months, with a projected 4-year survival rate of 6% [8]. Disease progression is intractably linked to a higher proviral load (PVL) in infected patients. Epidemiological, clinical, and molecular studies have shown that the expansion of HTLV-1-infected cells in vivo mainly occurs through the cellular replication of infected cells [9,10], and, as a result, HTLV-1 presents with very low antigenic variability. Since viral antigens elicit vigorous humoral and cell-mediated immune responses, reducing the expression of viral antigens is a critical step for virus survival in an infected host. There is a constant dynamic balance between virus-infected cells eliminated by the host immune system and the expansion of infected cells via cellular proliferation and de novo infection. Both cytotoxic T-lymphocytes (CTLs) and natural killer cells (NK) play critical roles in controlling HTLV-I virus expansion and the PVL in vivo. In support of these observations, several studies have reported the rapid occurrence of ATL in HTLV-I-infected individuals after immune-suppressive treatment following transplantation. Finally, the adoptive transfer of Tax-specific CTLs is sufficient to prevent HTLV-I-associated disease in animal models [11,12].

The HTLV-1 provirus exhibits several properties not seen in animal oncogenic retroviruses. The c-terminus of the provirus genome contains a region known as pX with at least five open reading frames (ORFs) encoding for regulatory proteins involved in viral pathogenesis [13,14,15]. The viral Tax (transactivator of the X region) protein specifically interacts with the cAMP response element (CREB) and acetyltransferase coactivators CBP/p300 (CREB-binding protein) and PCAF (p300/CREB-binding-protein-associated factor) to stimulate the expression of viral mRNA transcripts from the viral LTR (long terminal repeats) [16,17,18,19,20]. Tax is a potent modulator of transcription that can activate or repress many cellular genes involved in growth and survival [21] and is the driving factor behind the HTLV-1 random mutagenesis transformation model [22]. A matter of debate is whether Tax expression is needed in transformed ATL cells. On one hand, a common and striking feature of ATL cells is the apparent absence of detectable Tax expression in half of patients, suggesting that Tax expression may not be required in fully transformed cells. While the 5′ LTR is methylated in ATL patient samples, these results were obtained from global analyses and did not account for possible temporal and spatial differences. These results are confounded by possible differences in different ATL tumor clones within a patient and require analyses at the single-cell level. Whether Tax is or is not expressed in ATL cells has been controversial, but published evidence leaves little doubt that Tax expression is indeed present and required in ATL cells. The sporadic on/off switching of HTLV-1 Tax bursts of expression is essential to maintain the whole population of virus-induced leukemic cells. Tax is indeed expressed in a minor fraction of leukemic cells at any given time and the knockdown of Tax rapidly induces apoptosis in most cells, indicating that Tax is critical for maintaining the population, even if its short-term expression is limited to a small subpopulation [23]. Studies confirmed that primary ATL cells from patients with acute or chronic ATL express very low levels of Tax [24]. However, inhibition of Tax expression results in the reversal of nuclear factor-κB (NF-κB) activation, p53/promyelocytic leukemia protein (p53/PML) activation, and apoptosis [24]. In contrast, suppression of HTLV-1 bZIP factor (HBZ) expression in ATL cells was associated with increased Tax expression but did not affect the growth or the survival of ATL cells, suggesting that Tax is the cornerstone of HTLV-1 transformation [24]. Along these lines, the anti-sense transcript HBZ which is frequently expressed in ATL cells may serve to control Tax expression to optimal levels.

### Overview of the Molecular Mechanisms Associated with Tax-Induced Cellular Immortalization and/or Transformation

Although it is possible to immortalize (IL-2 (interleukin-2) ligand-dependent) or transform (ligand-independent) human primary T cells with HTLV-1 in vitro [25,26,27], most cell lines have been established by co-cultivation with lethally irradiated virus-producing cells rather than direct expansion of ATL tumor cells in vitro. In addition, the immortalization of primary T cells with viral Tax only occurs after a long culture period of over 8–10 months and is very inefficient; indeed, only one Tax-immortalized human T cell line has been reported in the literature [28]. Studies have shown that an autocrine mechanism may play a role in the growth of some HTLV-1-infected T cells in vitro, and the specific cytokine receptor may vary from cell line to cell line. Tax has been shown to activate the promoter for IL-2 and the IL-2Rα chain [29,30,31,32]. Similarly, transcription of IL-15 and the IL-15Rα chain is increased by Tax through the activation of the NF-κB pathway [33,34]. Furthermore, in the chronically infected HUT102 cell line, the expression of IL-15 and IL-15Rα is upregulated, supporting the notion that an autocrine mechanism of growth is important during some stages of infection [33]. Indeed, anti-IL-2R antibodies have been shown to reduce the tumor burden in NOD/SCID (nonobese diabetic/severe combined immunodeficiency) mouse models and ATL patients in vivo [35]. Previous investigations have demonstrated that Tax alone is unable to achieve full transformation of human primary T cells, referred to as growth in the absence of exogenous IL-2 [28]. In contrast, both human and murine fibroblasts have been transformed with Tax. 

The reasons behind these observations can be attributed to general differences between these cell types. In general, primary fibroblasts have a higher proliferative capacity compared to primary CD4 T cells, which may make them more amenable to the sustained cell division associated with cellular transformation. CD4 T cells are subject to stringent control mechanisms to avoid uncontrolled proliferation, making them generally less permissive to transformation. Finally, fibroblasts are relatively undifferentiated, and their transformation might require fewer obstacles related to differentiation status when compared to primary CD4 T cells. Although Tax appears to be a weak oncogene, it is indispensable to the virus transformation potential. 

Transduction of Tax and Tax mutants into primary T cells and immortalization assays have revealed a selection process characterized by several distinct crises with intermittent, excessive cell death followed by active proliferation [28]. We can speculate that some of these steps are related to pathways that are activated in HTLV-1-transformed ATL cells, such as the inactivation of tumor suppressors such as p53 and FBXW7 (F-box/WD repeat-containing protein 7); activation of specific oncogenic signaling pathways such as JAK/STAT (Janus kinase/signal transduction and transcription activation), PI3K (phosphatidylinositol 3-kinase), and NOTCH (notch receptor 1); activation of anti-apoptotic pathways; genetic and epigenetic alterations associated with genome instability; and finally the reactivation of hTERT (human telomerase reverse transcriptase) expression. While Tax can directly affect some of these events, others are the result of the in vitro selection of clones that have acquired specific genetic mutations. Inactivation of tumor suppressors and cell cycle checkpoints is often the result of the direct actions of Tax [22]. This results in unchecked cellular proliferation, creating a conducive environment for transformation. For example, the absence of cell cycle arrest in HTLV-I-transformed cells in vitro, despite an elevated level of p21WAF/CIP (cyclin-dependent kinase inhibitor 1), could be attributed to the phosphorylation of p21WAF/CIP by PI3K/AKT (protein kinase B), resulting in cytoplasmic retention and inactivation of p21WAF/CIP [36]. Therefore, Tax may, through its activation of PI3K/AKT, regulate p21WAF/CIP activity. In addition, Tax can directly interact with cyclin-dependent kinase 4 (CDK4) to form a complex capable of phosphorylating and inactivating Rb protein (retinoblastoma 1) to stimulate active cell cycle proliferation [37,38]. Tax directly inhibits cyclin-dependent kinase inhibitors to stimulate progression through the cell cycle and uncontrolled proliferation [39]. 

Activation of the JAK/STAT signaling pathway is a hallmark of HTLV-1 transformation [40] and represents a promising therapeutic target [41,42,43]. It is not clear how JAK/STAT is activated in ATL cells. While frequent mutations have been found in STAT3 [44], a high frequency of activating mutations in JAKs has not been reported. The most likely model for JAK activation in ATL cells is through hormone and cytokine receptor(s) signaling, resulting in JAK aggregation in lipid rafts at the cell membrane. Constitutive activation of the JAK pathway was associated with loss of src homology 2 (SH2)-containing phosphatase (SHP1) expression [45,46]. A direct implication of Tax in the activation of JAK/STAT has not been experimentally demonstrated. However, it is likely that, at least in the initial stages, Tax-mediated NF-κB activation results in the elevated expression of a myriad of cytokines and their receptors and indirect activation of JAKs. Activation of other oncogenic pathways such as PI3K/AKT and NOTCH is also found in HTLV-1-transformed cells [47,48,49,50]. In contrast to JAK/STAT, Tax has been shown to directly affect these pathways and to play a role in Tax-mediated fibroblast transformation [51]. Tax binds to PDZ domain-bearing proteins, including DLG-1 (discs large MAGUK scaffold protein 1), hScrib (scribble planar cell polarity protein), and MAGI-1 and MAGI-3 (membrane-associated guanylate kinase inverted −1 and −3) via its C-terminal PDZ-binding motif [52,53,54]. A physiological consequence of these interactions is Tax competition with PTEN (phosphatase and tensin homolog) for binding to DLG-1 [55]. Experimental data demonstrate that Tax induces the activation of the PI3K-Akt-mTOR (mechanistic target of rapamycin kinase) pathway by causing the mislocalization of the tumor suppressors PTEN and PHLPP (PH domain and leucine-rich repeat protein phosphatase) from the plasma membrane. Interestingly, the authors demonstrated that forced membrane localization of either PTEN or PHLPP was sufficient to prevent the negative effects of Tax on their phosphatase activity for PI3K [55]. The effects of Tax are likely important in the early steps of HTLV-1 transformation; however, it is unclear how PTEN is inactivated in transformed ATL cells in the absence of Tax expression. While early studies proposed a loss of PTEN expression in ATL cells, analyses of fresh uncultured samples demonstrated that PTEN is expressed at normal levels in ATL cells [49]. Studies have also shown that Tax can act downstream of the PI3K pathway by inducing the phosphorylation of AKT at Ser473 and Thr308 residues, resulting in the activation of AKT and subsequent inactivation of GSK3β (glycogen synthase kinase 3 beta) [56]. Another important oncogenic pathway in lymphoid cells is the NOTCH pathway. While activating genetic mutations have been found in the PEST (sequences enriched in proline (P), glutamate (E), serine (S), and threonine (T)) domain of the transcriptionally active NOTCH intracellular domain (NICD) and ubiquitin ligase FBXW7, a direct role of Tax has also been reported. Studies have shown that Tax increases the half-life of NICD, enhances the association of NICD with RBP-jκ (recombination signal binding protein for immunoglobulin kappa J region), and forms a ternary complex to stimulate transcriptional activation of NOTCH genes targets [57]. The stimulating effects of Tax on proliferation are clear shortly after its transfer to primary cells; however, after several days, these effects wane and are masked by an increase in cell death and permanent cell cycle arrest. In the sections below we will review the published literature and discuss both the pro- and anti-apoptotic functions of Tax, and how this integrates into its transforming abilities.

## 2. HTLV-1 Tax Control of the Apoptosis Cell Death Pathways

### 2.1. Activation of Apoptotic Pathways by Tax

The establishment of stable Tax-expressing cell lines in vitro is notoriously difficult and has been elusive, since continuous culturing of Tax-expressing cells results in negative selective pressure leading to loss of expression or cell death. In contrast, in vivo Tax transgenic models have been associated with a broad range of tumors from fibroblasts to hematopoietic B- and T cells. However, to our knowledge, none of these tumors have been able to be grown in vitro, suggesting that either Tax can sustainably be expressed only in primary cells and/or that the tumor microenvironment (TME) is required to sustain the proliferation and survival of Tax-expressing cells. Experimental studies have shown that Tax is a potent inducer of apoptosis. Factors that influence cellular fate in response to Tax are the cell type, the level of Tax expression, and the duration of Tax expression. Because Tax is highly immunogenic, its apoptotic-inducing functions can be viewed as a mechanism used by the virus to select infected clones with low suboptimal expression of Tax sufficient to evade immune surveillance while allowing Tax-mediated immortalization. Animal models of Tax-transgenic mice display increased apoptosis in in vivo tumors, which correlates with elevated expression of c-Myc, JUN (AP-1 transcription factor subunit), and p53 in Tax-expressing cells [58]. In vitro, Tax induces caspase-dependent cell death that correlates with the ability of Tax to regulate p300/CBP activity and not NF-κB. Experimental data showed that nuclear expression of the minimal CREB-binding protein (CBP)/p300-binding domain of Tax triggered an apoptotic death signal in both short-term clonal analyses as well as in transient cell death assays [59]. In HTLV-1-transformed cells that do not express detectable Tax protein, USP10 (ubiquitin-specific peptidase 10) inhibits stress-induced reactive oxygen species (ROS) production and apoptosis. However, in Tax-expressing cells, USP10 anti-apoptotic functions are blocked by Tax [60]. The dynamics of gene expression revealed by time-lapse imaging revealed a close correlation between Tax-induced apoptosis and alterations in the expression levels of IL-8 (interleukin-8), SMAD3 (SMAD family member 3), CDKN1A (cyclin-dependent kinase inhibitor 1), GADD45A and GADD45B (growth arrest and DNA damage-inducible alpha and beta), and IL-6 (interleukin-6). Given that these genes are associated with the regulation of stress kinase pathways, these data suggest that Tax might in part trigger apoptosis by activating genes linked to stress-response signaling pathways [61]. Consistent with this idea, over-expression of Tax sensitizes cells to DNA-damaging agents and triggers p53-independent apoptosis [62,63]. Several studies have also shown the effects of Tax on downstream events of death receptor (DR) signaling. Tax induces caspase-dependent cell death in Jurkat cells expressing CD95 (Fas) [64]. Additional studies showed that Tax expression in human T cells was associated with a significant increase in the expression of the Fas ligand (FasL) gene. This was significant because the induction of apoptosis by Tax was blocked upon interruption of the Fas/FasL signaling pathway [65]. Interestingly, fibroblasts expressing Tax are protected from apoptosis triggered by serum deprivation but are susceptible to tumor necrosis factor-alpha (TNFα)-mediated apoptosis. These observations indicate that Tax has distinct effects on cell death, contingent upon the cell type and the apoptotic stimulus [66].

### 2.2. Anti-Apoptotic Functions of Tax

**Figure 1:**
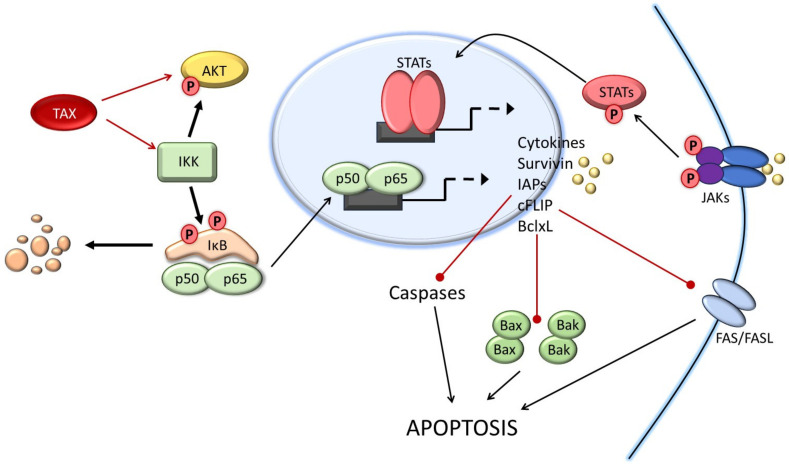
Control of apoptosis pathways by HTLV-1 Tax. Tax-mediated activation of the IKK complex results in IκB degradation and nuclear translocation of RelA/p65 to activate the expression of pro-survival genes. Increased expression of survivin and IAPs (inhibitors of apoptosis) efficiently inhibits caspases while cFLIP inhibits death receptor FAS/FASL signaling. In addition, BclxL can translocate to the mitochondria where it blocks Bax- and Bak-mediated release of cytochrome C and activation of caspases. Activation of NF-κB is also associated with the release of cytokines resulting in an autocrine/paracrine activation loop engaging receptor tyrosine kinase and activation of the JAK/STAT pathway. Tax also prevents pro-apoptotic functions of p53.

The section above highlights how high levels of Tax expression are associated with cell death; however, Tax is also a potent activator of cellular transduction pathways and induces the constitutive activation of major cellular pro-survival pathways NF-κB and PI3K [30,56] (Figure 1). 

These observations suggest that depending upon the level of Tax expression, the cell type, and the signaling molecules present in the microenvironment, the balance can be tipped from death to survival and vice versa. In vivo and ex vivo studies demonstrate the importance of Tax expression for the survival of HTLV-1-transformed ATL cells [24,67]. Numerous in vitro studies have demonstrated that Tax expression is associated with the production and release of numerous cytokines and growth factors with anti-apoptotic activities, listed below and summarized in Table 1 (IL-2, IL-15, IL-10, IL-6, IL-8, IL-1α, IL-5, IL-4, IL-13, IL-17, IL-12, IL-9, IL-21) [24,68,69,70,71,72,73,74,75,76,77,78,79]. Tax activates the expression of cytokine receptors such as IL-2R [29,30], IL-15R [34], IL-6R [80], IL-21R [79], and IL-17R [81]. In addition, Tax also stimulates the expression of various chemokines and chemokine ligands such as IFN-γ (interferon-gamma) and TNF-α [77], GM-CSF (colony stimulating factor 2) [82], MIP-1α/CCL3 (C-C motif chemokine ligand 3) [83], MIP-1β/CCL4 (C-C motif chemokine ligand 4) [84], MIP-3 (C-C motif chemokine receptor 7) [85], MCP-1 (C-C motif chemokine ligand 2) [86], CCL5/RANTES(C-C motif chemokine ligand 5) [87], CCL1 (C-C motif chemokine ligand 1) [88], CCL22 (C-C motif chemokine ligand 21) [89], CXCR7 (atypical chemokine receptor 3) [90], and CCR9 (C-C motif chemokine receptor 9) [91].

Tax activates the NF-κB pathway through several different mechanisms. Tax can directly interact with the IKKγ (inhibitor of nuclear factor kappa B kinase regulatory subunit gamma) to stimulate the IKK complex, triggering the continual phosphorylation and ubiquitin-mediated degradation of IκB to allow NF-κB RelA/p65 translocation to the nucleus and activation of canonical NF-κB1. Alternatively, Tax can form a complex with the p100 NF-κB precursor protein along with IKKα/IKKβ to facilitate the cleavage of p100 into the active p52 NF-κB subunit, resulting in the activation of the non-canonical NF-κB2 [92]. Some studies suggest that Tax-mediated NF-κB activation may result in opposite outcomes depending upon the cell dividing state. This notion may in part be explained by the effects of Tax on cellular DNA replication (see below). In human actively dividing KIT-225 cells, Tax was associated with growth arrest and cell death in a RelA/p65 canonical-dependent NF-κB pathway. In proliferative cells expressing Tax, inactivation of the non-canonical NF-κB and p38 MAPK (mitogen-activated protein kinase 1) pathways released Tax-mediated apoptosis, suggesting a role for Tax-NF-κB2-p38 MAPK axis in apoptosis. By contrast, in resting cells, Tax-mediated activation of RelA-NF-κB1 was required to prevent apoptotic cell death [93]. The activation of canonical NF-κB by Tax has been associated with increased expression of anti-apoptotic factors such as BclxL (BCL2 like 1), survivin, and Bfl-1(BCL-2-related protein A1) [86,94,95,96]. Another important anti-apoptotic factor in HTLV-1-transformed ATL cells is myeloid cell leukemia 1 (MCL1) protein. MCL1 protein stability is largely controlled by the ubiquitin ligase FBXW7, which is frequently inactivated in HTLV-1-transformed cells [50,97]. Furthermore, Tax-induced activation of IKK and TRAF6 (TNF receptor-associated factor 6) results in K63 ubiquitination of MCL1, thereby protecting it from genotoxic stress-induced degradation and enhancing its anti-apoptotic functions [98]. Experimental evidence in vitro demonstrates that Tax-induced cell death can be efficiently prevented by co-expression of B-cell lymphoma 2 (Bcl-2) [59,99]. Tax can increase c-FLIP (FADD-like anti-apoptotic molecule 1) protein expression through the Tax-IKK-NF-κB signaling pathway, providing resistance against death receptor-mediated apoptosis, such as FasL and TNF-related apoptosis-inducing ligand (TRAIL) [100]. Interestingly, Tax-triggered autophagy depends on the activation of IKK but not the activation of NF-κB RelA/p65 [100]. This phenomenon is reminiscent of Tax’s ability to stimulate telomerase activity through the IKK complex independently of transcriptional activation [101]. 

In addition to the upregulation of pro-survival proteins, Tax may also confer apoptosis resistance by suppressing the expression or activities of pro-apoptotic factors or signaling pathways. For instance, Tax has been shown to suppress the expression of the pro-apoptotic BH3-only proteins Bim (BCL2-like 11) and Bid (BH3-interacting domain death agonist) by enhancing the expression of hypoxia-inducible factor-1α (HIF-1α) [102]. Enforced expression of Bim or Bid reverted resistance to CD95- and TRAIL-induced apoptosis of Tax-expressing cells [102]. Another example is the pro-apoptotic factor Bcl-2-associated X protein (Bax). Transcription of the Bax gene expression is controlled by p53 and strongly activated in response to chemotherapeutic DNA damaging agents. Tumor suppressor p53 is a central regulator inducing cell cycle arrest and apoptosis in response to DNA damage. However, in HTLV-1-transformed ATL cells, p53 functions are inactivated. Furthermore, several laboratories have demonstrated that Tax efficiently inhibits p53 transcriptional activities [103,104,105,106]. Other functions of Tax such as CREB activation also play a role in Tax-mediated protection from cell death. Under serum starvation conditions, Tax protein protects cells from apoptosis by preventing cytochrome c release and Bax relocation to the mitochondria [107].

## 3. Context-Dependent Expression of Tax Induces Senescence or Immortalization

### 3.1. Tax Overexpression Is Associated with Rapid Stress-Induced Senescence (SIS) in Pre-Transformed p53-Deficient Cells 

**Figure 2:**
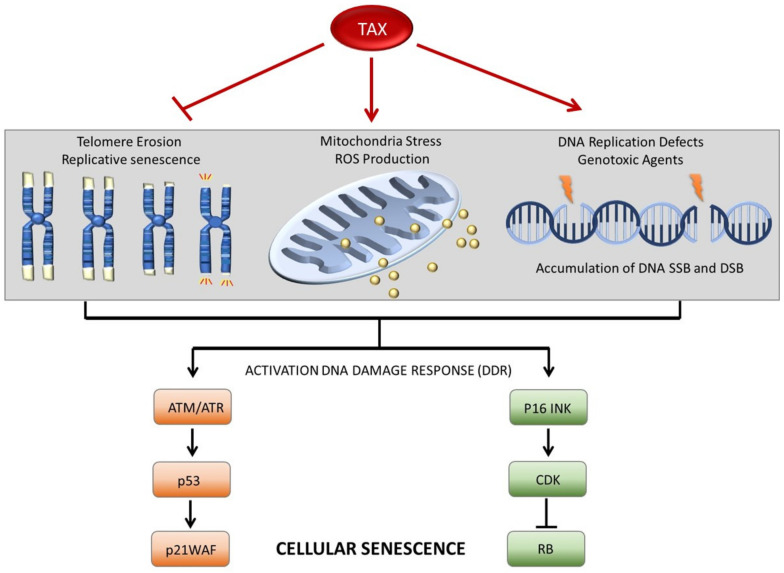
Cellular senescence pathways affected by Tax. Schematic representation of different physiological processes associated with induction of cellular senescence. While cellular division is associated with telomere shortening and replicative senescence, Tax can reactivate hTERT expression and stimulate its activity. These effects are sufficient to prevent telomere-induced foci (TIF) and activation of the DDR. Tax expression is associated with rapid cell proliferation and various metabolic stress progression, such as the production of reactive oxygen species (ROS). In turn, ROS induces DNA damage and activation of ATM/p53 response. Tax expression impairs DNA replication fork, resulting in stalling and fallout, creating an accumulation of double DNA strand breaks and activation of the DDR. Activation of the DDR can stimulate ATM/p53 or p16INK pathways, leading to irreversible cellular senescence.

Cells that are subjected to stress signals can undergo apoptosis or enter permanent cell cycle arrest and senescence. Different types of stress signals have been characterized, such as those produced by oncogene-induced senescence (OIS) [108], chronic inflammation, and therapy-induced senescence (TIS). TIS is a type of cellular senescence caused by the accumulation of DNA double-strand breaks (DSBs) in cancer cells and is most common during cancer chemotherapy treatment [109]. Expression of Tax has been shown to interfere with the cellular DNA repair machinery. Tax has been shown to prevent nucleotide excision repair (NER) in part through increasing PCNA (proliferating cell nuclear antigen) gene expression [110,111], to inhibit base-excision repair (BER) of oxidative damage [112], and to block homologous recombination (HR) [113]. How Tax interferes with the HR repair pathway is currently unknown, but some studies suggest that Tax forms speckled structures (TSS), which recruit and sequester 53BP1 (tumor protein p53 binding protein) upon ATM (ATM threonine/serine kinase) signaling, thereby preventing 53BP1 binding to DDSBs [114]. In addition, Tax has been shown to interact with Chk1 (checkpoint kinase 1), resulting in premature attenuation of the ATM/Chk1 signaling axis in Tax-expressing cells [115]. Several studies have also shown that Tax expression not only blocks DNA repair but is associated with the accumulation of DNA strand break foci and increased phosphorylation of H2AX [113,116] (Figure 2). 

A direct implication of Tax in the formation of DDSBs was demonstrated by molecular combing techniques showing that DNA replication forks encounter difficulties when replicating complex DNA, resulting in slower progression and more frequent pauses or stalls in the presence of Tax expression. Tax-related replication defects can be partially mitigated by an upsurge in the activation of backup origins of replication but at the expense of DDSBs [117]. The status of p53 may play a major role in deciding the fate between apoptotic death and senescence, and in p53-deficient cells, Tax overexpression may be associated with a similar physiological cellular response as described for TIS. Additional studies have demonstrated that Tax-mediated NF-κB activation induces the formation of R-loops [118]. Like the effects of Tax on DNA replication, R-loops can also interfere with DNA replication, resulting in replication fork collapse and formation of DDSBs [119]. Although Tax-mediated NF-κB activation plays a central role in the secretion of cytokines that stimulate cellular proliferation, excessive cytokine signaling can result in chronic inflammation and increased expression of p21WAF/CIP and p27KIP (cyclin-dependent kinase inhibitor 1B), leading to cellular senescence [120,121]. In turn, senescent cells can produce senescence-associated secretory phenotype (SASP) vesicles, which can stimulate inflammation in their tumor microenvironment. Studies have shown that transient overexpression of Tax-mediated activation of IKKα and RelA/p65 results in OIS in transformed epithelial cells deficient for p53 such as HeLa [121,122]. Because of the nature of HeLa cells, it is difficult to assess the physiological relevance of these data, and confirmation of these results in primary T cells is needed.

### 3.2. Tax Expression in Primary Cells Is Associated with the Reactivation of hTERT and Stability of Telomere Ends

Even in the absence of stress signal-induced senescence, each cellular replication is associated with a progressive shortening of the telomeres in normal cells, eventually resulting in the activation of the replicative senescence program and a permanent irreversible cell cycle arrest (Figure 2). Replicative senescence restricts the proliferative capacity and blocks the accumulation of mutations and genomic defects that are responsible for the initiation and progression of cellular transformation. In cancer cells, preservation of sufficient telomere ends and unlimited cellular replication is ensured by the reactivation of hTERT, or the activation of a pathway known as the alternative lengthening of telomeres (ALT). Reactivation of telomerase activity has been associated with the development of HTLV-1-induced leukemia [123,124]. Indeed, activation of the hTERT promoter by Tax-induced NF-κB was demonstrated by transduction into human primary lymphocytes [125]. In addition, transcriptional activation of the hTERT promoter by the PI3K pathway and direct activation of hTERT enzymatic activity by Tax stimulation of IKK suggests multiple levels of control to preserve telomere end integrity [48,101]. 

## 4. Discussion and Perspectives

**Figure 3:**
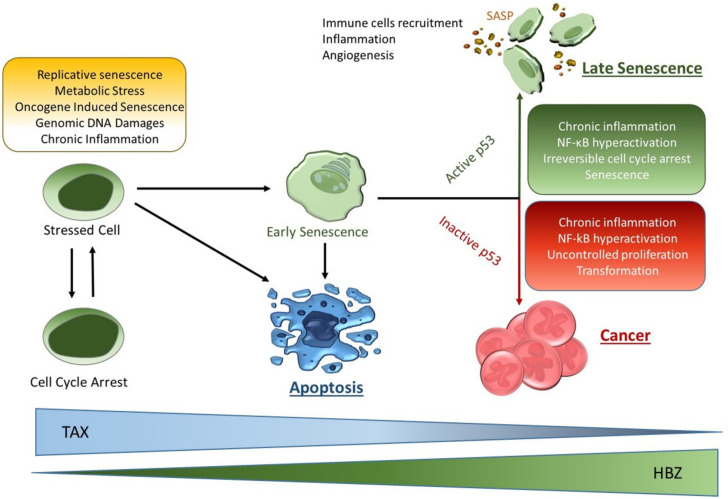
Schematic representation of cellular fates in response to various stress stimuli. Evolution of infected cells relative to the levels of Tax and HBZ expression. Upon stress signals, induced by replicative senescence, metabolic stress, high expression and oncogene-induced senescence, genotoxic agent exposure, and genomic DNA damage or chronic inflammation, the cell may undergo transient cell cycle arrest to repair and/or prevent accumulation of more cellular damages. If cell cycle checkpoints are not functioning properly or the damage is too extensive to be repaired, the cell may undergo apoptosis. Cells that undergo early senescence may progress to the irreversible late senescence stage in the presence of an active p53 signaling pathway or transformation if p53 is inactivated.

This review describes some of the opposing functions of the HTLV-1 virus Tax protein in cellular death and transformation. For 30 years, Tax has been at the center of HTLV-1 research and there is no doubt that Tax is a critical factor for virus replication and pathogenesis. Although the expression of Tax in vivo has been a matter of debate, recent evidence demonstrates that Tax is indeed expressed in ATL cells in vivo [23,24,126], and required for tumor cell expansion and survival. Targeting Tax expression has been found to provide benefits in pre-clinical models [67,127,128,129]. In HTLV-1 infected patients, CTLs directed against the Tax protein play a major role in controlling the expansion of virus-infected cells and maintaining a low proviral load. Impaired CTL responses in patients with high PVL compared with asymptomatic patients with low PVL support the notion that efficient control of HTLV-I in vivo depends upon the quality rather than the quantity of the CTL response [130,131]. Therefore, understanding how the virus prevents CTL activity is essential. Indeed, the findings that infected cells with extremely low levels of Tax expression can be targeted by high-avidity CTLs in vitro raises the possibility of developing therapeutic vaccines for the treatment of HTLV-I-associated diseases [132]. Notably, the adoptive transfer of Tax-specific CTLs is sufficient to prevent HTLV-I-associated disease in animal models [133]. Because of its low antigenic variability, the virus has evolved multiple strategies to avoid high expression of Tax and unmasking of infected cells to the hosts’ immune system. Along these lines, Tax-induced apoptosis may be regarded as a self-defense mechanism used by the virus to fight against the host immune response. The fact that Tax uses several different strategies to induce cellular apoptosis in newly infected cells exerts a selective pressure against elevated Tax expression and the acquisition of mutations in the pro-apoptotic pathway. This allows an overall increase in cell survival. These events favor selection and emergence of tumor clones with low Tax expression. In addition, it is possible that in some infected cells, the high level of Tax stimulates apoptosis to generate apoptotic bodies carrying virus particles [134]. This allows the virus to spread the infection to the monocytes/macrophages that are attracted by the apoptosis-induced microenvironment and inflammation. Additional viral factors may control Tax expression. The 5′LTR is frequently methylated in ATL patient samples, thereby reducing viral transcription. However, previous studies have shown that in the presence of phobol esters, Tax can effectively activate transcription from methylated 5′ LTR [135]. The virus also encodes regulatory proteins that can suppress Tax activities. HTLV-1 p30 is a nucleolar non-shuttling protein capable of interacting with and blocking the nuclear export of Tax/rex mRNAs, thus reducing Tax expression and virus replication [136,137]. Another viral regulatory protein, p13, has been found to interact with Tax and reduces recruitment of the CBP/p300 transcriptional co-activator by Tax, thereby dampening transcriptional activities [138]. Finally, the HTLV-1 viral HBZ factor has been shown to prevent both Tax-mediated cell death and Tax-induced senescence [122,139]. These effects of HBZ are associated with the silencing of Tax mRNA expression by HBZ anti-sense mRNA rather than some inherent function(s) of HBZ. Furthermore, HBZ has been shown to interact with CREB and prevent the formation of the ternary complex Tax/CREB/CBP on the 5′LTR, thereby reducing the expression of viral mRNAs [140]. In HTLV-1-transformed ATL cells, HBZ expression is likely required to keep Tax under control for provirus expression. Although the induction of senescence is widely regarded as a tumor suppressor defense mechanism, it also comes with risks (Figure 3). 

Indeed, in vivo senescent cells that are not eliminated may play a role in preventing new tissue and promoting the proliferation, migration, invasiveness, and epithelial–mesenchymal transition (EMT) of tumors [141,142]. Senescent cells produce SASP that can stimulate tissue remodeling and angiogenesis, thereby facilitating the growth and spread of precancerous cells (Figure 3). In addition, while SASP factors may attract immune cells to eliminate potential threats, this may in certain instances create an inflammatory environment conducive to tumor growth. Given the multiple roles of HTLV-1 in both transformation and senescence, combination therapy including senolytic agents may be a promising approach for future anti-cancer therapy in HTLV-1 ATL patients.

## Figures and Tables

**Table 1:** Summary of signaling and apoptosis genes regulated by HTLV-1 Tax. References are provided in the main text.

	Effects of Tax	Mechanism of Activation
Cytokines		
IL-1α	activated	NF-κB
IL-2	activated	NF-κB/NF-AT
IL-4	activated	NF-κB/NF-AT/NF-IL6
IL-6	activated	NF-κB
IL-8	activated	NF-κB
IL-9	activated	NF-κB/IRF4
IL-10	activated	NF-κB
IL-12	activated	NF-κB
IL-13	activated	NF-AT/GATA3
IL-15	activated	NF-κB
IL-17	activated	CREB/ATF
IL-21	activated	NF-κB/AP1
Cytokine Receptors		
IL-2R	activated	NF-κB/NF-AT
IL-6R	activated	?
IL-15R	activated	NF-κB/IRF-4
IL-17R	activated	?
IL-21R	activated	AP1/IRF
Chemokines/Ligands		
IFN-γ	activated	IL-12
TNF-α	activated	NF-κB
GM-CSF	activated	NF-κB/CREB/ATF/NF-IL6
MIP-1α/CCL3	activated	NF-κB
MIP-1β/CCL4	activated	NF-κB
MIP-3	activated	NF-κB
MCP-1	activated	NF-κB
CCL5/RANTES	activated	NF-κB
CCL1	activated	?
CCL22	activated	?
CXCR7	activated	NF-κB
CCR9	activated	?
Apoptosis Regulators		
Bcl-xL	activated	NF-κB/CREB/ATF
Survivin	activated	NF-κB
Bfl-1	activated	NF-κB/cJun/JunD
MCL-1	activated	TRAF6
c-FLIP	activated	NF-κB
Bim	suppressed	HIF-1α
Bid	suppressed	HIF-1α
Bax	suppressed	TP53
